# Radiotherapy-induced isolated left main coronary artery disease presenting with cardiogenic shock: A case report

**DOI:** 10.1097/MD.0000000000029116

**Published:** 2022-04-22

**Authors:** Bo Li, Yuan Liu, Zhiyang Lou, Weihua Zhang, Mingyou Zhang, Quan Liu

**Affiliations:** Department of Cardiovascular Medicine, The First Hospital of Jilin University, Changchun, China.

**Keywords:** cardiovascular damage, case report, fibrous proliferation, Hodgkin's lymphoma, mediastinal radiotherapy

## Abstract

**Rationale:**

Mediastinal radiotherapy is a common practice for treating breast cancer and Hodgkin's lymphoma. Radiotherapy causes cardiovascular damage and has attracted increasing attention, particularly among Hodgkin's lymphoma patients, as they receive a higher dose of radiation.

**Patient concerns:**

A 36-year-old woman with a past medical history of Hodgkin's lymphoma presented with persistent chest pain for 3 hours. She experienced exertional chest pain 1 month before when she was climbing stairs, which disappeared after a few minutes with rest, but recurred with a similar level of exertion. Three hours before admission to the emergency room, the chest pain persisted and was accompanied by diaphoresis and dyspnea.

**Diagnosis:**

Cardiogenic shock caused by radiotherapy-induced left main coronary artery disease.

**Interventions:**

Urgent angiography revealed left main coronary artery stenosis. Intravascular ultrasonography showed diffuse fibrous proliferation in the left main coronary artery. Hemodynamic instability was resolved after drug-eluting stent implantation.

**Outcomes:**

The patient was discharged uneventfully 5 days after the procedure, with a prescription for dual antiplatelet and statin therapy. She was asymptomatic with good exercise tolerance at the 3-month follow-up.

**Conclusion:**

Radiotherapy-induced isolated left main coronary artery disease is a rare complication of cancer radiotherapy and can occur years or decades after treatment. Fibrous proliferation is a characteristic pathologic change in the exposed coronary arteries.

## 1 Introduction

Radiotherapy is an effective treatment for many malignant tumors, especially Hodgkin's lymphoma.^[1]^ However, it has been known to cause radiotherapy-induced heart disease. Radiotherapy-induced isolated left main coronary artery disease is a rare complication of Hodgkin's lymphoma radiotherapy.^[2]^ The development of radiotherapy-induced coronary artery disease may take years or decades before becoming symptomatic. The ostial left or right coronary artery are frequently involved, with variable clinical presentation from stable angina to acute coronary syndrome. Fibrous proliferation is a characteristic pathologic change in the exposed coronary artery.^[3]^ This report aimed to outline the management of a patient with radiotherapy-induced left main coronary artery disease presenting with cardiogenic shock. This study was approved by the ethics committee of The First Hospital of Jilin University, and written informed consent was obtained from the patient for publication of this manuscript.

## 2 Case presentation

A 36-year-old woman with a past medical history of Hodgkin's lymphoma, presented with exertional chest pain for 1 month. She reported exertional chest pain upon climbing stairs 1 month ago, which disappeared a few minutes after rest, but recurred with exertion. Three hours prior to admission to the emergency room, the chest pain persisted and was accompanied by diaphoresis and dyspnea.

The patient was seen acutely stressed, and vital signs showed a heart rate of 136 beats per minute, blood pressure of 80/58 mm Hg (1 mm Hg = 0.133 kPa), respiratory rate of 30 breaths per minute, and oxygen saturation of 97% while breathing ambient air. She reported worsening dyspnea in the supine position, but her neck veins were not distended and cardiac and pulmonary auscultation was normal. There was no edema in the lower limbs.

She was diagnosed with Hodgkin's lymphoma 5 years ago. She was treated with 6 cycles of combined chemotherapy with doxorubicin, bleomycin, vincristine, and dacarbazine; 2 cycles of combined chemotherapy with bleomycin, etoposide, doxorubicin, cyclophosphamide, vincristine, procarbazine, and prednisone; followed by 1 cycle of radiotherapy to her mediastinum for a total dose of 4140 cGy (100 cGy = 1 Gy). No signs of tumor relapse were observed in the past 5 years during regular follow-up. She has no history of hypertension, diabetes, or hyperlipidemia, never smoked, and had no family history of cardiovascular disease.

**Figure 1:**
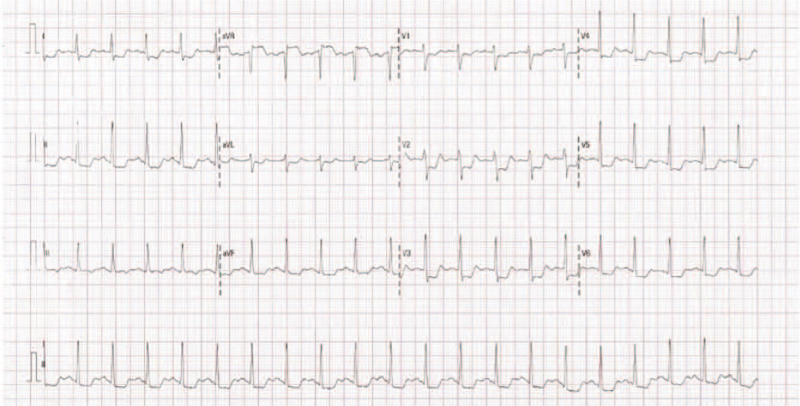
Electrocardiogram in the emergency room shows ST-segment elevation in the aVR lead and ST-segment depression in the V1-V6 leads, indicating left main coronary artery disease.

**Figure 2:**
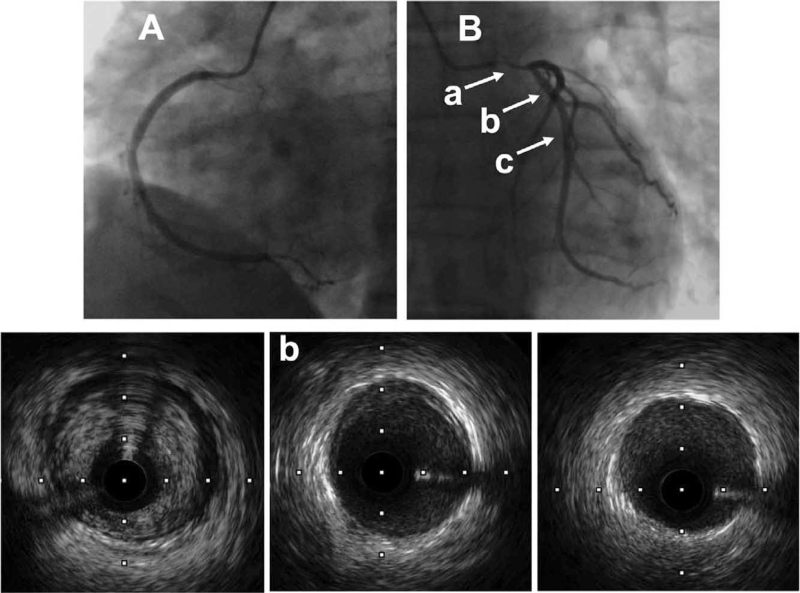
Urgent angiography shows a normal right coronary artery (A) and 80% to 90% stenosis of the left main coronary artery (B). The corresponding intravascular ultrasound cross-sectional image shows an eccentric homogenous fibrous plaque at the area of stenosis (a), and normal structure of the coronary artery distal to the lesion (b, c).

The admission electrocardiogram showed ST-segment elevation in the aVR lead and significant ST-segment depression in the V1-V6 leads, indicating a left main lesion (Fig. 1). Urgent coronary angiography revealed 80% to 90% stenosis in the ostium of the left main coronary artery, extending to the bifurcation. The distal vessels and right coronary artery angiographically appeared normal (Supplementary Digital Content movies S1, S2). Intravascular ultrasound revealed an eccentric fibrous lesion in the left main coronary artery (Fig. 2), without lipidic plaque or calcium. The luminal area of the ostial left main artery was 2.81 mm^2^. The artery distal to the left main lesion was normal, without any signs of atherosclerotic plaque. due to the absence of classic atherosclerotic risk factors, and typical presentation which consistent with the Radiotherapy-induced coronary artery stenosis, diagnosis of Radiotherapy-induced isolated left main coronary artery stenosis was established.

The lesion was successfully treated with placement of a 3.5 × 20 mm drug-eluting stent, and her chest pain was relieved after the procedure. Laboratory results showed serum myoglobin was 229.00 ng/mL, peak cardiac troponin I was 2.48 ng/mL, and B-type natriuretic peptide was within the normal range. Echocardiography revealed left ventricular ejection fraction of 60%, absence of segmental myocardial wall movement, and no pericardial effusion. She was discharged uneventfully 5 days after the procedure with a prescription of dual antiplatelets and a statin. She was asymptomatic with good exercise tolerance at 3-month follow-up.

## 3 Discussion

Radiotherapy-induced coronary artery disease is a common cause of morbidity and mortality in patients treated with radiotherapy for malignant tumors. The development of radiotherapy-induced coronary artery disease may take years or decades before becoming symptomatic. Overlapping conventional cardiovascular risk factors can promote the development of coronary artery disease (CAD), and complicate changes in lesion pathophysiology. However, observational studies have shown that this population typically experiences sudden death and ischemic heart disease-associated deaths, even with few conventional cardiovascular risk factors.^[3,4]^

Radiotherapy-induced pathological changes in the coronary arteries are different from those in typical atherosclerosis. First, the radiotherapy-induced lesion usually does not cause systemic inflammation proven by irradiated animal models, and out-of-field arteries were unaffected.^[5]^ Second, histologically, the lesions in the exposed artery are diffuse with prominent fibrosis proven by autopsy.^[4]^ Even in the high-fat diet hyperlipidemic apolipoprotein E knockout mice model, prominent fibrosis with a smaller lipid core was observed.^[6]^ This was consistent with the IVUS findings in our case and the literature.^[7,8]^ Atherosclerotic lesions, especially advanced lesions, are characterized by a necrotic core with a large lipid pool, which can be unstable even before becoming obstructive. Thus, thrombotic complications due to plaque rupture or plaque erosion often bring patients to the clinician with the most dramatic presentations, such as acute coronary syndrome. Fibrin-rich plaques are the characteristic pathologic changes of radiotherapy-induced coronary artery lesions, which are more stable than atherosclerotic plaques and tend to progress in a more chronic and predictable process. Patients who present with suspected ischemic symptoms and a history of radiation therapy should undergo CAD screening, even without risk factors for atherosclerosis.

Observational studies have shown that patients with radiotherapy-induced CAD have worse outcomes than atherosclerotic CAD^[9,10]^ Both percutaneous coronary intervention (PCI) and coronary artery bypass grafting have been proposed as treatments. However, data comparing PCI and coronary artery bypass grafting for the treatment of radiotherapy-induced CAD are limited. Dehiscence and infections due to poor wound healing caused by radiation exposure are of concern. Expert consensus suggests PCI as a first-line treatment for patients without valvular or severe pericardial disease.^[11]^

In conclusion, we report a case of radiotherapy-induced left main coronary artery disease presenting with cardiogenic shock. Intravascular ultrasonography confirmed a fibrous lesion in the left main coronary artery. Hemodynamic stabilization was achieved by primary PCI, with good short-term follow-up results. Radiotherapy causes cardiovascular damage and has attracted increasing attention. Further studies on the mechanism, prevention, and treatment of radiotherapy-induced CAD are warranted.

## Acknowledgments

We thank the Elsevier author services office for their language editing services.

## Author contributions

**Conceptualization:** Weihua Zhang.

**Investigation:** Bo Li, Zhiyang Lou, Quan Liu.

**Methodology:** Yuan Liu.

**Project administration:** Weihua Zhang.

**Resources:** Yuan Liu.

**Validation:** Zhiyang Lou.

**Writing – original draft:** Mingyou Zhang.

**Writing – review & editing:** Weihua Zhang.

## Supplementary Material

Supplemental Digital Content

## Supplementary Material

Supplemental Digital Content
